# Capsules with bacteria and fungi in distinct compartments: A platform for studying microbes from different kingdoms and their cross-communication

**DOI:** 10.1371/journal.pone.0277132

**Published:** 2022-11-11

**Authors:** So Hyun Ahn, Amy J. Karlsson, William E. Bentley, Srinivasa R. Raghavan

**Affiliations:** 1 Department of Chemical & Biomolecular Engineering, University of Maryland, College Park, MD, United States of America; 2 Fischell Department of Bioengineering, University of Maryland, College Park, Maryland, United States of America; Yenepoya University, INDIA

## Abstract

Recently, we have created ‘artificial cells’ with an architecture mimicking that of typical eukaryotic cells. Our design uses common biopolymers like alginate and chitosan to create multi-compartment capsules (MCCs) via oil-free microfluidics. MCCs (~ 500 μm in diameter) can be engineered with multiple inner compartments, each with a distinct payload. This mimics the distinct organelles in eukaryotic cells, each of which has unique properties. In this study, we encapsulate microbial cells from two distinct kingdoms — *Pseudomonas aeruginosa* (bacteria) and *Candida albicans* (fungi) — in the inner compartments of MCCs. The two microbes are commonly found in biofilms at sites of infection in humans. We first demonstrate that the MCC can serve as a simple platform to observe the comparative growth of the cells in real time. Unlike typical co-culture in solution or on agar plates, the cells can grow in their own compartments without direct physical contact. Moreover, the hydrogel matrix in the compartments mimics the three-dimensional (3-D) environment that cells naturally encounter during their growth. Small molecules added to the solution are shown to permeate through the capsule walls and affect cell growth: for example, cationic surfactants inhibit the fungi but not the bacteria. Conversely, low pH and kanamycin inhibit the bacteria but not the fungi. Also, when the bacteria are present in adjacent compartments, the fungal cells mostly stay in a *yeast* morphology, meaning as spheroidal cells. In contrast, in the absence of the bacteria, the fungi transition into *hyphae*, i.e., long multicellular filaments. The inhibition of this morphological switch in fungal cells is shown to be induced by signaling molecules (specifically, the quorum sensing autoinducer-1 or AI-1) secreted by the bacteria. Thus, the MCC platform can also be used to detect cross-kingdom signaling between the compartmentalized microbes.

## 1. Introduction

Cells are the basic building blocks of all life forms on earth. From unicellular (e.g. bacteria) to multicellular (e.g. plants, animals), every living organism consists of cells [1]. A defining characteristic of eukaryotic cells is the presence of many internal compartments (organelles) [1–3]. Each organelle is enclosed within a membrane, which regulates the entry and exit of molecules [1]. Thus, each organelle has different contents in its lumen and its membrane, and in turn serves different functions. In the last decades, researchers have attempted to replicate this multi-organelle architecture in synthetic microscale structures, which are termed ‘artificial cells’ or ‘protocells’ [4–11]. In this regard, our labs have recently reported cell-like structures that we refer to as *multi-compartment capsules* (MCCs) [11]. These MCCs (sizes ~ 500 μm) are made from biopolymers like alginate, and our method allows us to encapsulate different payloads in each compartment (sizes ~ 100 μm). Payloads with sizes of 5 nm or larger (including enzymes and nanoparticles) remain sequestered in the hydrogel matrix within each compartment, but small molecules can enter or exit the compartments.

A crucial advantage of our approach to MCCs is that they are made by an *oil-free* microfluidic method, with gas being used to shear off aqueous droplets as the fluid leaves a capillary tube [11]. This facilitates the encapsulation of biological cells in the compartments. In our initial study, we encapsulated two strains of bacteria in adjacent compartments of MCCs and monitored communication between the cells. One producer (P) strain received a chemical signal from the external medium and then secreted its own signaling molecules (autoinducer-2 or AI-2). AI-2 is one of a family of autoinducer molecules that mediate quorum sensing (QS) in bacteria [12, 13]. QS is the phenomenon by which bacteria alter their gene expression when they reach a ‘quorum’, i.e., when their cell density becomes sufficiently high [12]. In our experiment, the AI-2 diffused from one compartment into the adjacent one, where it turned on QS in the reporter (R) cells present there — i.e., induced the cells to express a fluorescent protein [11].

**Fig 1:**
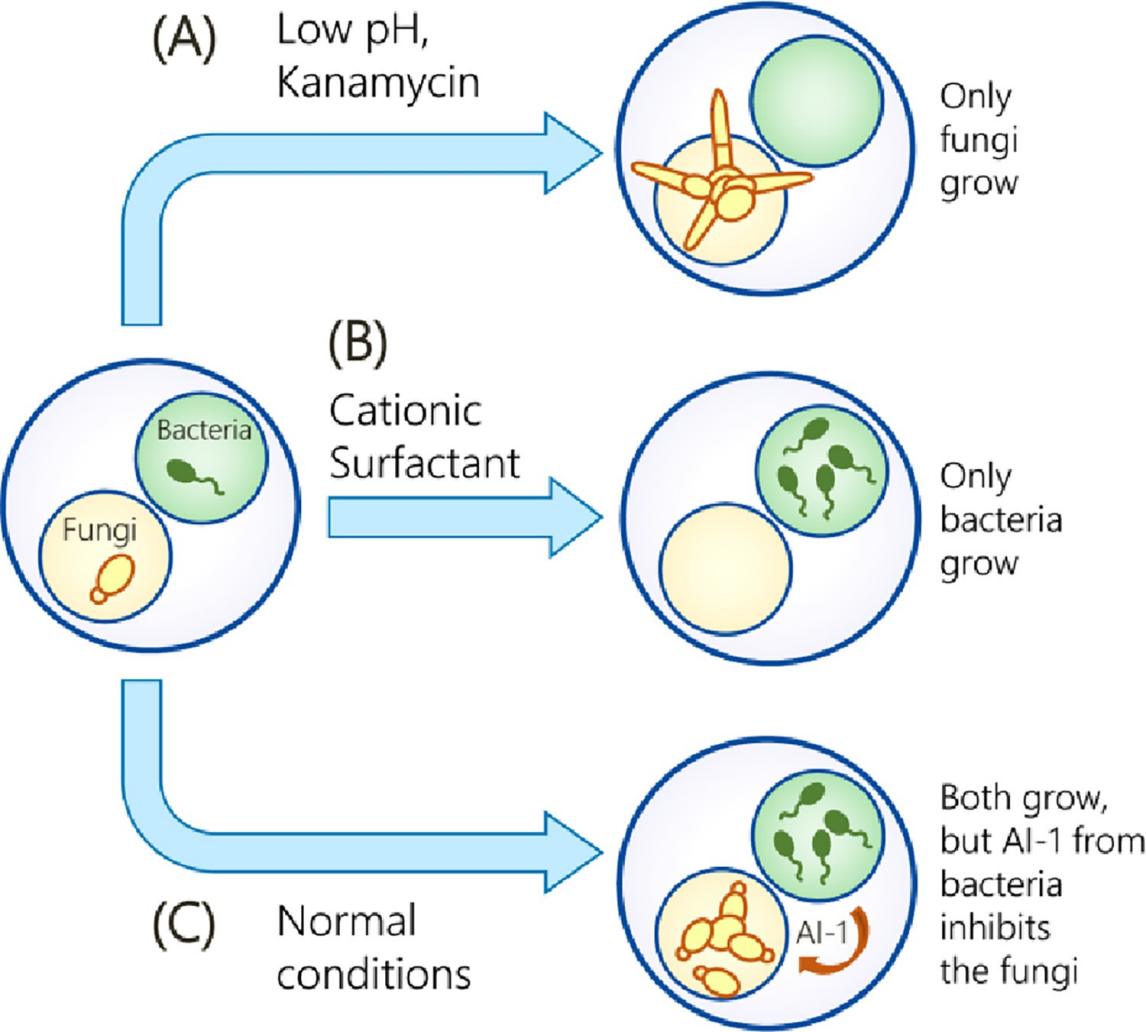
Overview of this paper. We synthesize multi-compartment capsules (MCCs) with fungi and bacteria in separate compartments. (A) When chemicals like kanamycin are added or when the pH is low, only fungi grow whereas the bacteria do not. (B) When other chemicals such as cationic surfactants are added, only the bacteria grow whereas the fungi do not. (C) Under normal growth conditions, inter-kingdom interaction is observed where signaling molecules like AI-1 produced by the bacteria affect the growth and morphology of the fungi. The fungi remain in yeast, not hyphal form.

In the present study, we extend the use of MCCs as a platform to study microbes from *different kingdoms*, specifically *bacteria* and *fungi*. As the bacterial species, we select the Gram-negative *Pseudomonas aeruginosa*, while the fungus we choose is *Candida albicans*. The two microbes are part of the normal human microbiota but are also opportunistic pathogens, i.e., they turn pathogenic as the opportunity presents, such as when the host has a weak immune system [14–16]. They are often found together in biofilms, including in over 70% of human infections involving biofilms [17], such as in patients with cystic fibrosis [15]. Co-cultures of *P*. *aeruginosa* and *C*. *albicans* are usually studied in liquid culture or by plating on the same agar plate [18–23]. Both methods have their disadvantages. In liquid co-culture, cells are in physical contact during their growth, which can affect their growth rates, although it is difficult to pinpoint how they affect each other [24]. On an agar plate, cells grow largely in 2-D at the air/agar interface, but this does not replicate the 3-D constraints they encounter in their natural habitats [24]. MCCs with a hydrogel scaffold offer a 3-D environment for cell growth, and by keeping the cells in separate compartments, we eliminate physical contact (Fig 1). We are able to monitor cell growth in real-time by optical microscopy and thus elucidate how different chemicals (e.g., surfactants, QS signaling molecules) or external conditions (e.g., pH) affect both kingdoms. Scenarios where the fungi grow at the expense of bacteria (Fig 1A) or vice-versa (Fig 1B) are both reported in this paper.

One further motivation for our studies is to explore cross-kingdom signaling between the microbes. QS molecules secreted by bacteria can affect cells from other kingdoms including fungi (and vice versa) [18–23, 25, 26]. Such signaling is often observed in biofilms, which are a matrix of polysaccharides and other components secreted by the microbes [17]. Biofilms in our body or on medical devices are often associated with adverse effects on health [14, 18]. Thus, there is a need to understand and mitigate biofilm growth. In biofilms, *P*. *aeruginosa* and *C*. *albicans* are known to have an antagonistic relationship, i.e., they inhibit each other [20, 25]. This relationship is mediated by QS molecules: e.g., *P*. *aeruginosa* cells produce autoinducer-1 (AI-1), which induce *C*. *albicans* cells to remain as ‘*yeast*’ (i.e., spheroidal cells) rather than as ‘*hyphae*’ (i.e., long multicellular filaments) [19, 27]. Generally, fungi like *C*. *albicans* are more invasive or virulent in their hyphal form [28]. In this study, we are able to monitor the morphology of *C*. *albicans* cells in real-time in our MCC construct (Fig 1C). The fungi switch from yeast to hyphae when *P*. *aeruginosa* cells are absent, but remain as yeast when these bacteria are present. Our studies collectively show that the MCC is a simple, yet versatile platform for simultaneously examining various cell types and also for interrogating the dynamics of cell-cell communication.

## 2. Results and discussion

### 2.1. Encapsulation of *C*. *albicans* and *P*. *aeruginosa* in MCCs

**Fig 2:**
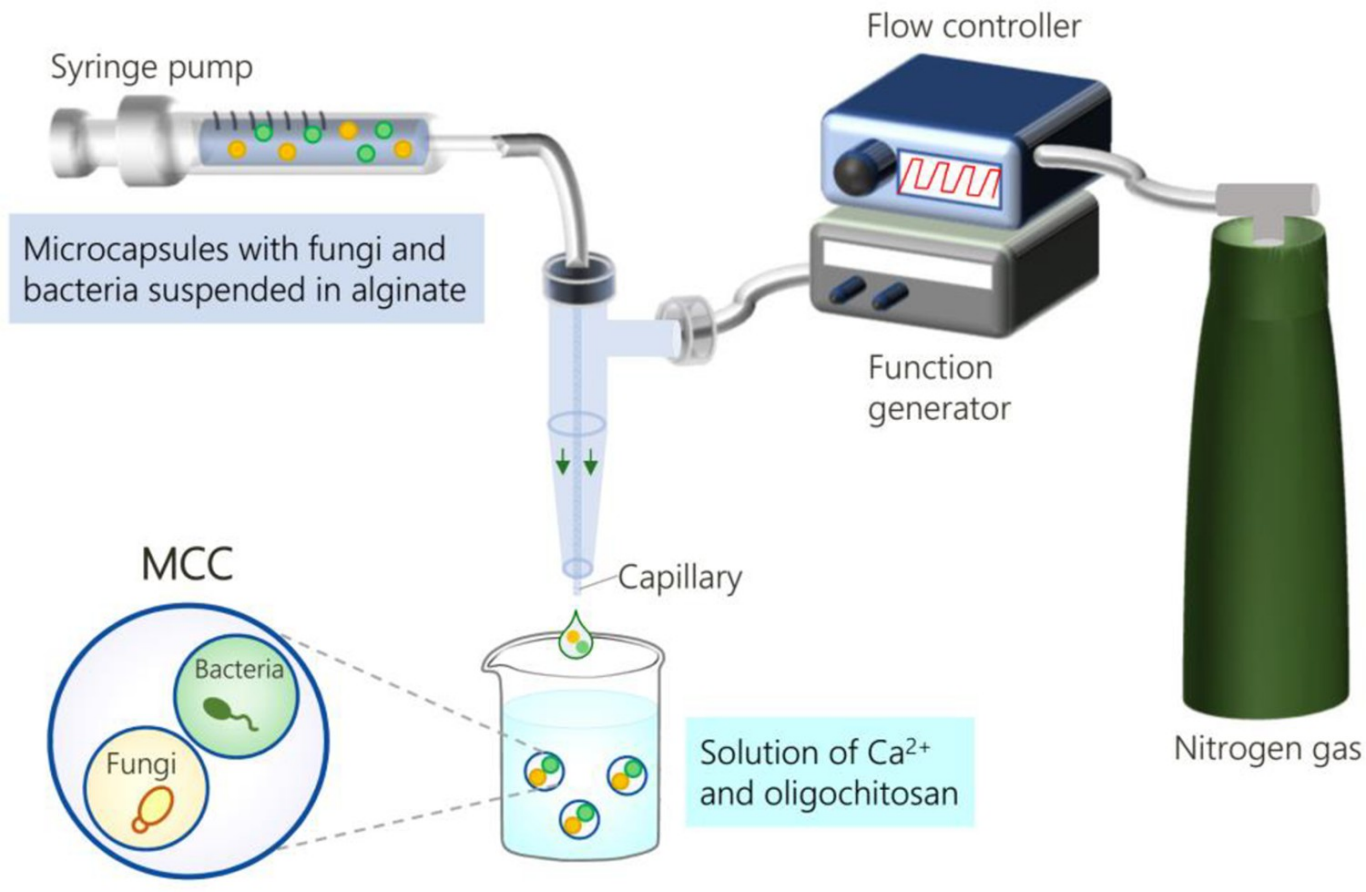
Microfluidic synthesis of MCCs with bacteria and fungi in distinct compartments. Microcapsules containing each microbe are made first. These are mixed with alginate and used as a feed for the MCCs. The feed is flowed through a 400 μm capillary and droplets are sheared off the capillary tip by pulses of nitrogen gas. The droplets are collected in the reservoir, where they are converted to MCCs due to crosslinking of the alginate by Ca^2+^ and chitosan.

The microfluidic setup shown in Fig 2 is used to synthesize MCCs with bacteria (*P*. *aeruginosa*) and fungi (*C*. *albicans*) in separate inner compartments. First, we make the microcapsules that will serve as inner compartments [11]. Suspensions of the bacteria and fungi (yeast form) in 2% alginate solutions are fed through a 150 μm capillary at a flow rate of 10 μL/min (see Experimental Section for further details). Nitrogen gas, pulsed at 1 Hz around the capillary tip, shears off droplets containing cells. which are collected in the reservoir solution (0.1 M CaCl_2_ + 1 wt% oligochitosan). The droplets are thereby converted to microcapsules with diameters ~ 200 μm over an incubation time of 30 min, after which they are washed and stored in phosphate-buffered saline (PBS). Note that in each microcapsule, the anionic alginate chains will be crosslinked by contact with the multivalent cations (Ca^2+^) as well as the cationic chains of the oligochitosan [11]. Also, because our microfluidic setup is oil-free, we can easily encapsulate viable microbial cells in the microcapsules.

Next, to make the MCCs, we prepare the feed by mixing microcapsules containing fungi and microcapsules with bacteria in an equal ratio in 1 mL of PBS combined with 4 mL of a 2% alginate solution. As shown in Fig 2, this suspension is fed by a syringe pump through a capillary with a diameter of 400 μm [11]. Droplets are again sheared off the capillary tip by nitrogen gas pulsed at 1 Hz. The droplets are collected in the same reservoir solution as above and allowed to incubate for 30 min, whereupon they are converted to MCCs with diameters ~ 500 μm. We focus on MCCs with two compartments, one with the fungi and the other with bacteria, as shown by the schematic. Because of their convenient size, MCCs of interest can be isolated from the population using a micropipette and placed in specific wells of a plate along with growth media for further studies.

### 2.2. Growth of fungi and bacteria under various conditions

**Fig 3:**
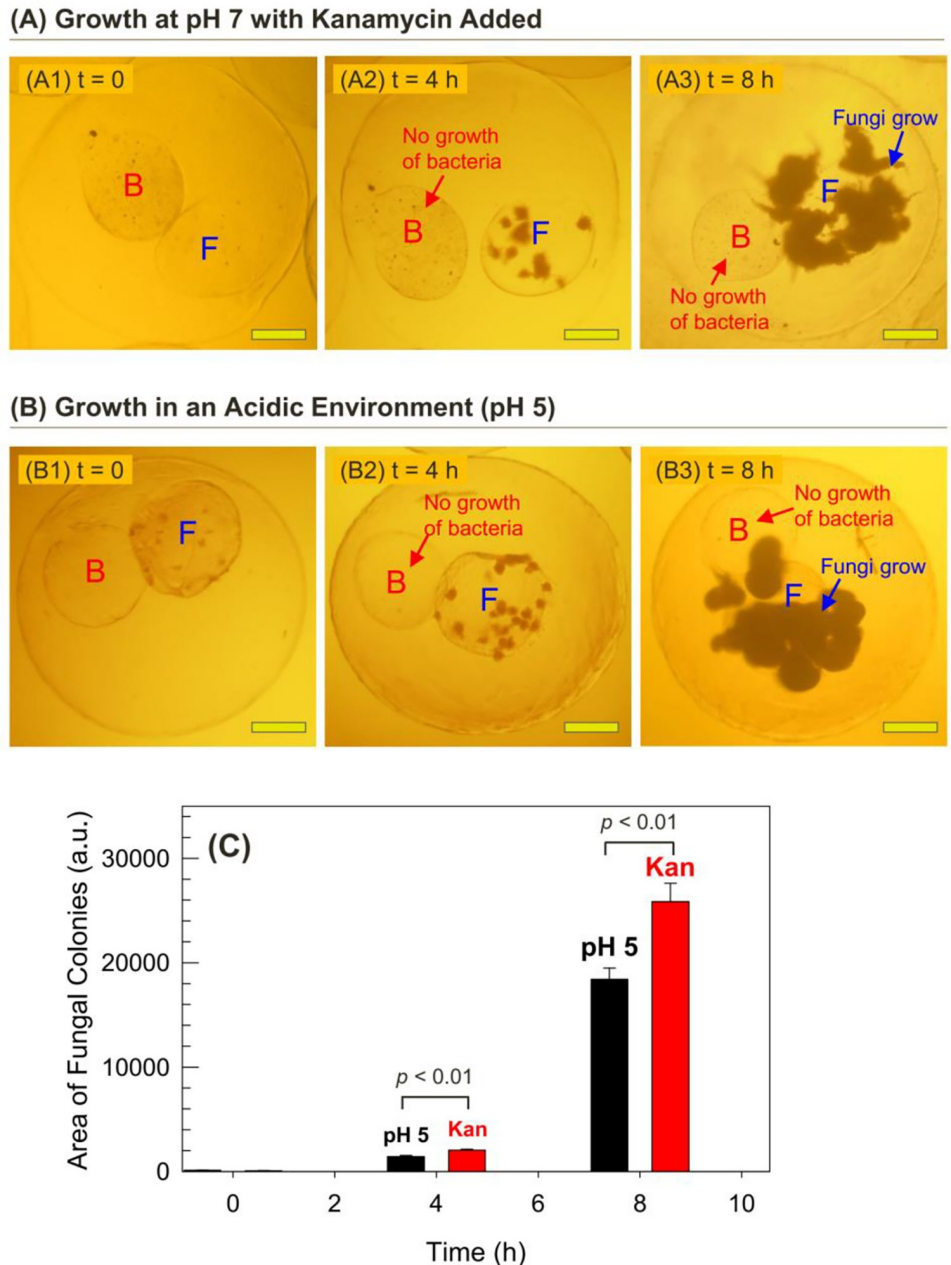
MCCs with fungi and bacteria showing preferential growth of the fungi under certain conditions. Optical micrographs at various time points of an MCC with Compartment F containing fungi (*C*. *albicans*) and Compartment B containing bacteria (*P*. *aeruginosa*). (A) The presence of 50 μg/mL kanamycin inhibits the growth of bacteria, but the fungi grow uninhibited. (B) When the pH is lowered to 5, again the fungi grow whereas the bacteria show no growth. (C) A graph showing the areas covered by fungal colonies at the 4 h and 8 h time points. The error bars correspond to standard deviations from n = 10 observations. (Scale bars in the images: 100 μm.).

Our MCC construct allows real-time observation of cell growth by optical microscopy. Because the fungi and bacteria are in separate compartments, their growth can each be monitored independently. If there is a change in the environment that affects either cells, the effects can be observed and quantified. In this regard, Fig 3 shows two cases where *P*. *aeruginosa* is affected by chemical additives much more than *C*. *albicans*. In Fig 3A, 50 μg/mL of kanamycin is added at *t* = 0 to the growth medium around the MCCs. Kanamycin is an aminoglycoside that kills bacteria by binding to their ribosomes and thus blocking protein synthesis [29]. However, kanamycin is expected to be ineffective against fungi as it does not bind to fungal ribosomes [29].

The results in Fig 3A focus on a typical MCC in the culture and show images of it at different time points. The MCC has two inner compartments, one with *C*. *albicans* (Compartment F) and the other with *P*. *aeruginosa* (Compartment B). Nanoparticles of carbon black (CB) are incorporated in trace amounts (~ 0.01%) along with the bacterial feed so that Compartment B can be distinguished in the images; note that it has a slightly darker hue at *t* = 0 than its counterpart (Image A1). No cell colonies are visible at this stage in either compartment. After 4 h, spheroidal colonies of fungi can be seen in Compartment F (Image A2), indicating robust fungal growth. However, no growth is observed in Compartment B. At the 8 h mark (Image A3), the fungi have grown further, and large colonies are seen both inside and outside Compartment F. The fungal colonies also show visible hyphae [28], i.e., thread-like filaments at their surfaces. Conversely, no growth is observed in Compartment B and this can be attributed to the antibacterial action of kanamycin.

Similar results are seen for the effects of acidic pH on the cells (Fig 3B). Typically, MCCs are cultured in growth medium at neutral pH (7.2 to 7.4) and under these conditions, within 3 h, small colonies are visible in Compartment F in a typical MCC (Image B1). Although not as clearly visible, the bacteria are also growing in Compartment B in the same MCC. At this point, which is *t* = 0 for our pH experiment, we add acetate buffer to the system, bringing the pH down to 5. Image B2, which is after 4 h at pH 5 shows growth of the fungi into spheroidal colonies, but no growth of the bacteria. Subsequently, at the 8 h mark (Image B3), there is still no growth of the bacteria in Compartment B, but the fungi have grown further and their colonies extend out of Compartment F. Interestingly, the fungal colonies at pH 5 show smooth surfaces with less filamentation (indicating that the cells are mostly in the yeast rather than hyphal form) compared to those grown at neutral pH in Fig 3A.

The pH effects observed in Fig 3B are consistent with the reported literature on *C*. *albicans* and *P*. *aeruginosa* [30, 31]. Specifically, *P*. *aeruginosa* is known to grow optimally at neutral pH. Acidic pH hinders growth because it lowers the activity of enzymes in the cells or damages proteins on cell membranes [30]. In contrast, fungi like *C*. *albicans* can grow in a wide range of pH conditions, including highly acidic conditions. Unlike bacteria, fungi have a built-in mechanism that allows them to pump extra H^+^ ions across the cell membrane and maintain neutral pH in their cells [31, 32]. These aspects explain why the fungi show robust growth in the MCCs at low pH whereas the bacteria do not.

Growth of fungi at both the conditions studied in Fig 3 can be quantified by image analysis using ImageJ. For this, we measured the areas of the fungal colonies in a given MCC at various time points, and in each case, we sampled at least *n* = 10 MCCs and computed the average. Once small colonies form in Compartment F (e.g., at the 4 h mark), the number of colonies remain the same, but each colony grows in size. When the colonies are large, they overlap on the image, making it difficult to resolve individual ones, which is why we prefer to focus on colony area rather than number. The data in Fig 3C show that the fungal colonies grow more at neutral pH (despite the presence of kanamycin) than at acidic pH. Also, between the 4 and 8 h mark, there is a 10-fold increase in colony area in both experiments.

**Fig 4:**
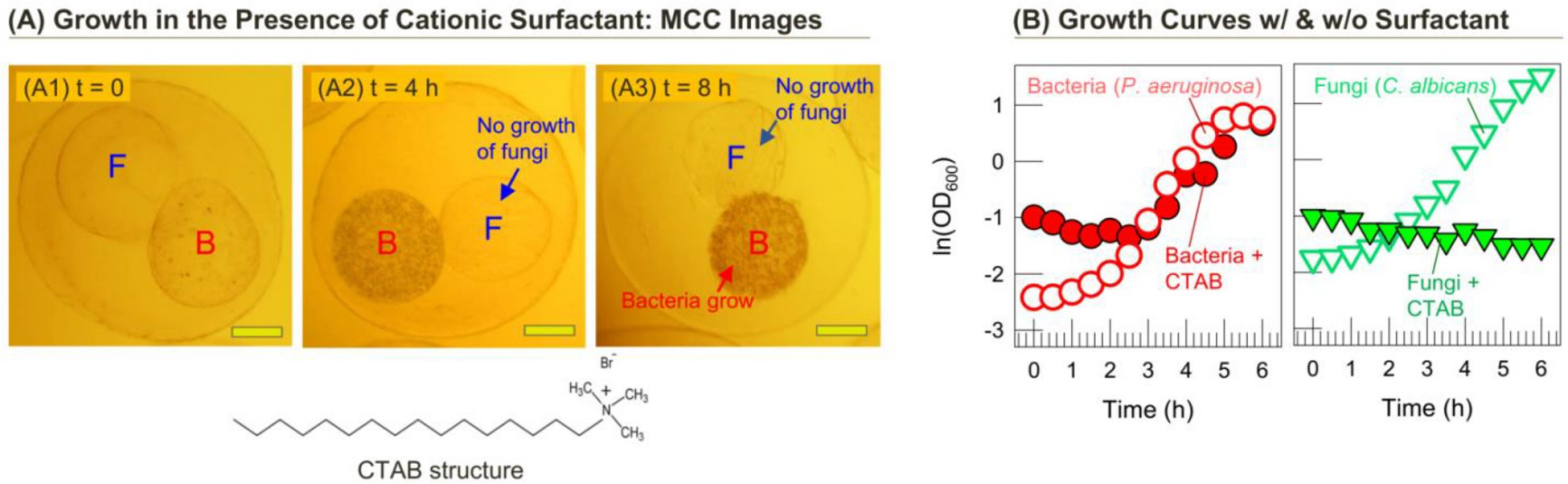
MCCs with fungi and bacteria showing preferential growth of the bacteria in the presence of a cationic surfactant. (A) Optical micrographs at various time points of an MCC with Compartment B containing bacteria (*P*. *aeruginosa*) and Compartment F with fungi (*C*. *albicans*). The MCC is cultured with 500 μM of the surfactant CTAB (structure shown). Growth is only observed in Compartment B while the fungi are killed by the surfactant. (B) Growth curves (semi-log plot of optical density (OD) vs. time) for *P*. *aeruginosa* and *C*. *albicans* cultures grown with and without 500 μM CTAB. The fungi show no growth when the surfactant is present. (Scale bars in the images: 100 μm).

Next, we demonstrate a scenario where the bacteria, but not the fungi, grow in the MCCs (Fig 4). In this case, we add the cationic surfactant cetyl trimethylammonium bromide (CTAB) at a 500 μM concentration to the culture medium at *t* = 0 and monitor cell growth. The images in Fig 4A focus on a typical MCC over time, and as before, the MCC has two inner compartments, one with *C*. *albicans* (Compartment F) and the other with *P*. *aeruginosa* (Compartment B). Surfactants are amphiphilic molecules with a hydrophilic head and a hydrophobic tail. In the case of CTAB (structure in Fig 4A), it has a cationic head and a C_16_ tail. Microbial cells are expected to have strongly anionic membranes, and as a result, cationic surfactants like CTAB will have a strong propensity to bind and embed in the membranes, thereby disrupting the membranes and causing cell lysis [33]. Indeed, CTAB is reported to have antiviral, antibacterial and antifungal properties [34].

Interestingly, however, in our experiment we find that CTAB affects only the fungi. Comparing Images A1 to A3 over an 8 h period in Fig 4, we see that *P*. *aeruginosa* grows undeterred in Compartment B, whereas no growth of *C*. *albicans* is observed in Compartment F. Evidently, CTAB binds to the fungal membranes and kills the fungi whereas it has no effect on the bacteria. The differential effects of CTAB are verified with liquid cultures (i.e., without capsules). Growth curves of *P*. *aeruginosa* alone with or without 500 μM CTAB are quite similar (Fig 4B), with the cells reaching a similar optical density (OD) over 6 h of culture at 37°C. In contrast, the growth curve of *C*. *albicans* in the presence of 500 μM CTAB remains flat (near-zero) whereas normal growth is seen in the absence of CTAB. Thus, CTAB is toxic to *C*. *albicans* but not to *P*. *aeruginosa*. The lack of effect of CTAB on *P*. *aeruginosa* is consistent with studies by other research groups on similar systems [35, 36].

### 2.3. Morphological transition of *C*. *albicans*

**Fig 5:**
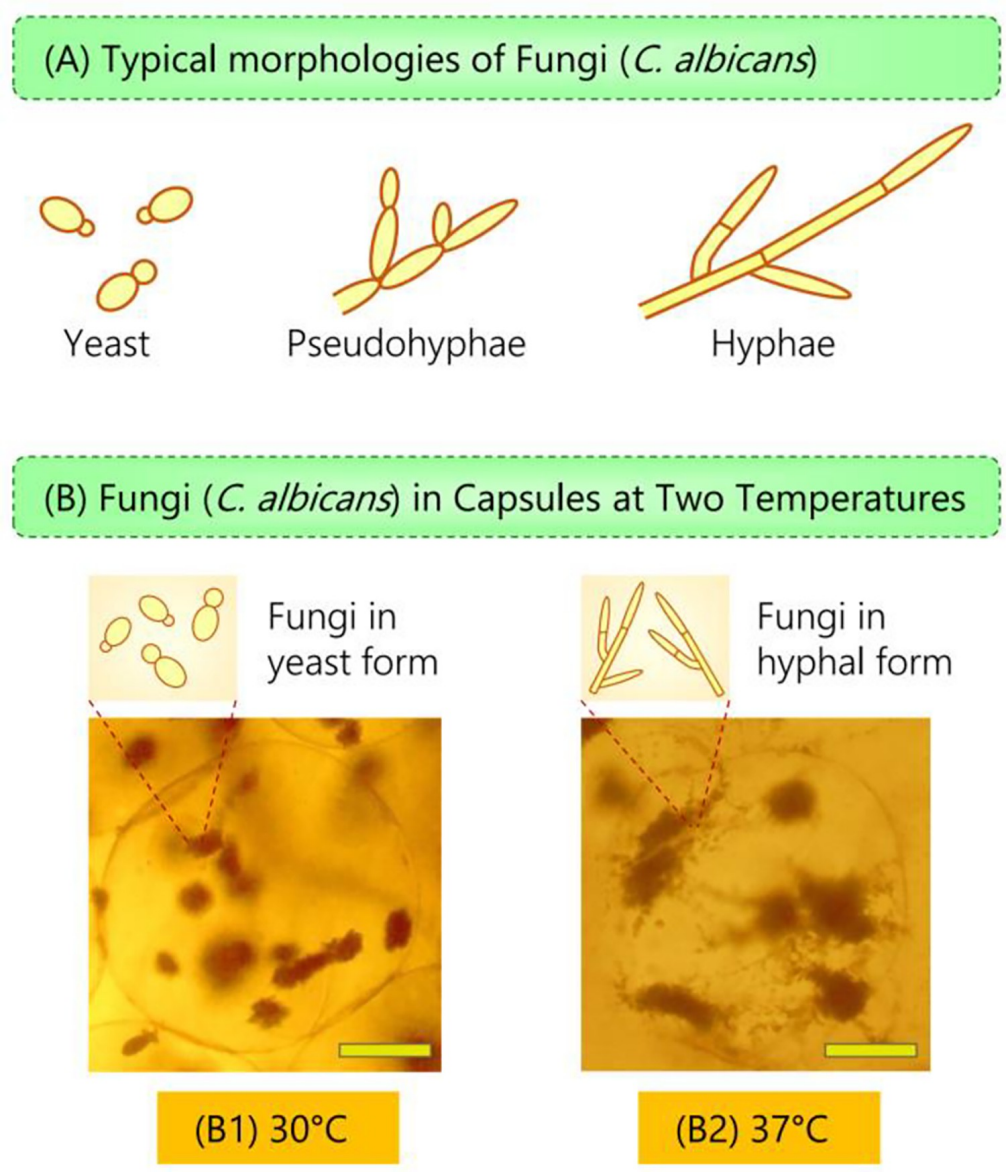
Morphology of *C*. *albicans* at different temperatures. (A) Schematics of typical fungal morphologies: as yeast, pseudohyphae, and hyphae (multicellular filaments). (B) Optical micrographs showing that *C*. *albicans* in capsules cultured at 30°C take on the yeast form whereas they transition to hyphae at 37°C. The images are taken after 12 h of culture. (Scale bars: 100 μm).

We had mentioned in the Introduction that fungi like *C*. *albicans* can grow as yeast or hyphae. The transition between these two morphologies is dictated by pH, temperature, and various nutrients or chemicals [28]. Here, we study the morphological transition of *C*. *albicans* as a function of temperature (Fig 5). When microcapsules with these fungi are incubated at 30°C, the cells are in the yeast form. The growing cells form dense clusters (colonies) (Fig 5A), which are spheroidal or ellipsoidal in shape and have relatively ‘smooth’ surfaces. At 37°C, however, a significant number of cells transform into multicellular filaments, i.e., hyphae. The cells still form colonies at 37°C, which continue to have a dense core, but numerous hyphae emanate from the surfaces of the colonies, giving them a ‘rough’ appearance.

**Fig 6:**
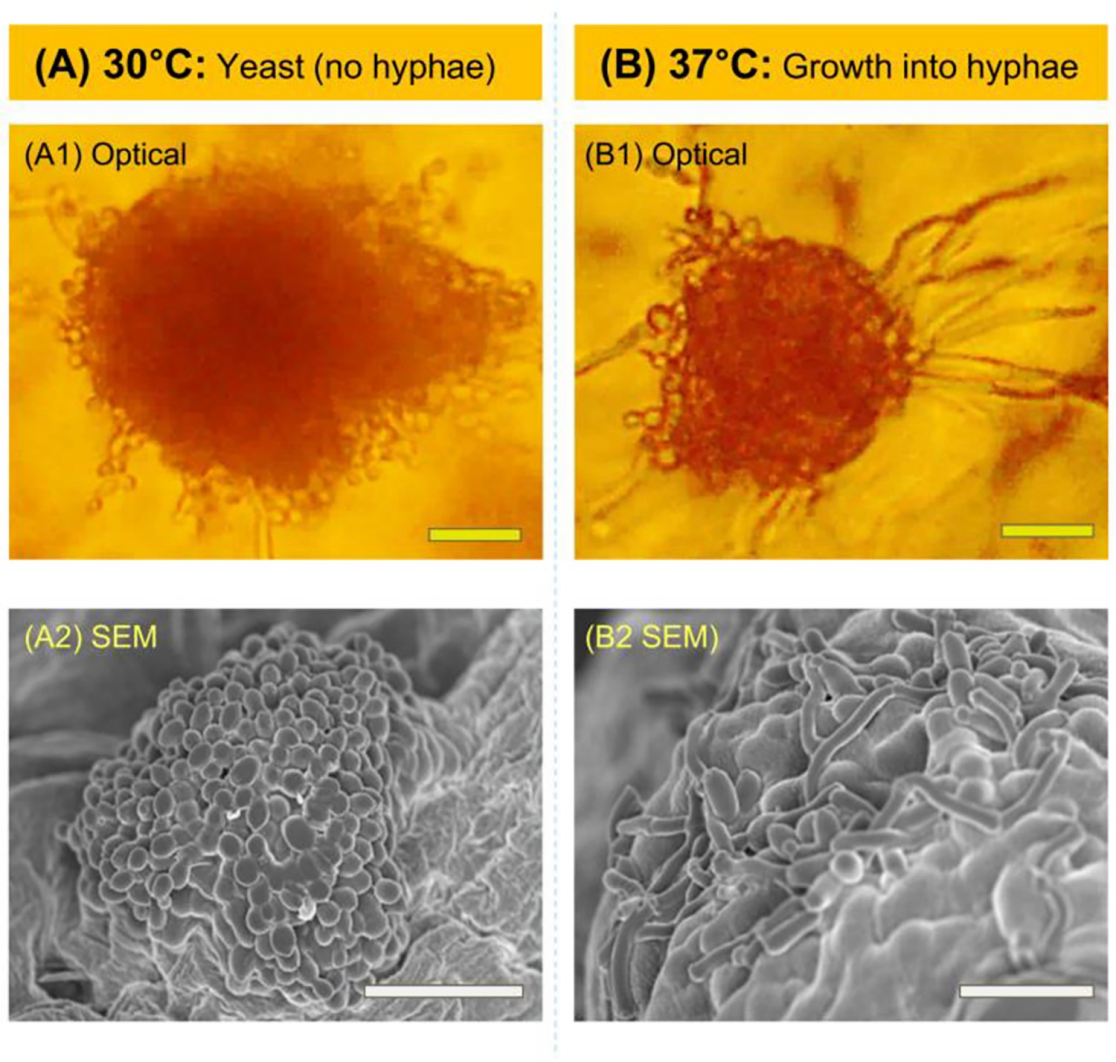
Optical and SEM micrographs of a single *C*. *albicans* colony in yeast and hyphal forms. (A) At 30°C, the cells remain in yeast form and few filaments are seen. The SEM shows that the colony is a cluster of multiple ellipsoidal cells. (B) At 37°C, the cells transition to hyphae and long filaments grow in random directions from the colony surface. (Scale bars: 20 μm).

To visualize these differences more clearly, Fig 6 shows close-up images from optical and SEM microscopy of single colonies. At 30°C (Fig 6A), the growing colony does have ‘rough’ surfaces, indicating that cells are adding onto the colony at its outer boundaries, but most of the cells are small and discrete, i.e., not filaments (Image A1). The SEM (Image A2) clearly shows that the colony is a cluster of close-packed yeast cells. Both the individual cells as well as the colony are spheroidal. At 37°C (Fig 6B), the morphology is very different. Long filaments (i.e., hyphae) [28] extend out of the colony from its surfaces in all directions (Image B1). In the SEM (Image B2), the cells composing the outer surface of the colony are elongated filaments, not spheroidal yeast cells. Note that the filaments are collapsed on the colony in the SEM image because the sample has to be dried for SEM imaging.

### 2.4. Cross-Kingdom signaling between fungi and bacteria in an MCC

**Fig 7:**
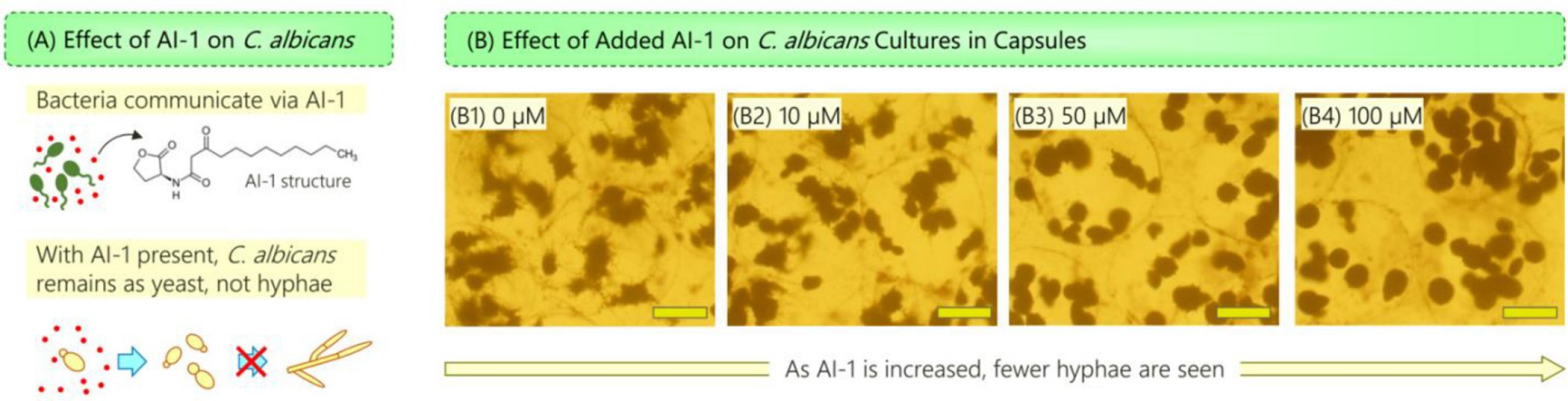
Effect of QS molecule (AI-1) produced by *P*. *aeruginosa* on *C*. *albicans*. (A) Schematics showing that as *P*. *aeruginosa* grow, they communicate by producing AI-1, which inhibits the transition of *C*. *albicans* from yeast to hyphae. (B) Morphology of encapsulated *C*. *albicans* cultured in the presence of different AI-1 concentrations. With no AI-1, hyphae are formed. With increasing AI-1, hyphae are inhibited. (Scale bars: 100 μm).

In the Introduction, we mentioned the antagonistic interaction between *P*. *aeruginosa* and *C*. *albicans* in biofilms. This interaction is believed to be mediated by the QS molecule AI-1, as depicted schematically in Fig 7A. AI-1 is expected to inhibit the transition of *C*. *albicans* from yeast to hyphae [19, 27]. To verify this effect, we cultured *C*. *albicans* in microcapsules and added synthetic AI-1 to the culture medium. Fig 7B shows images of capsules at 37°C after 12 h of culture (note that this temperature was found to be suitable for hyphal growth in Figs 5 and 6). With no AI-1 present, the cells do exhibit robust growth with hyphae extending out from all the colonies (Image B1). With 10 μM of AI-1, the hyphae are much reduced compared to the control case (Image B2). Increasing the AI-1 to 50 μM (Image B3) and then to 100 μM (Image B4) further reduces the hyphae. In Image B4, the colonies mostly have a spheroidal shape with no hyphae around their surfaces. These results confirm the expected effects of AI-1 on the fungi.

**Fig 8:**
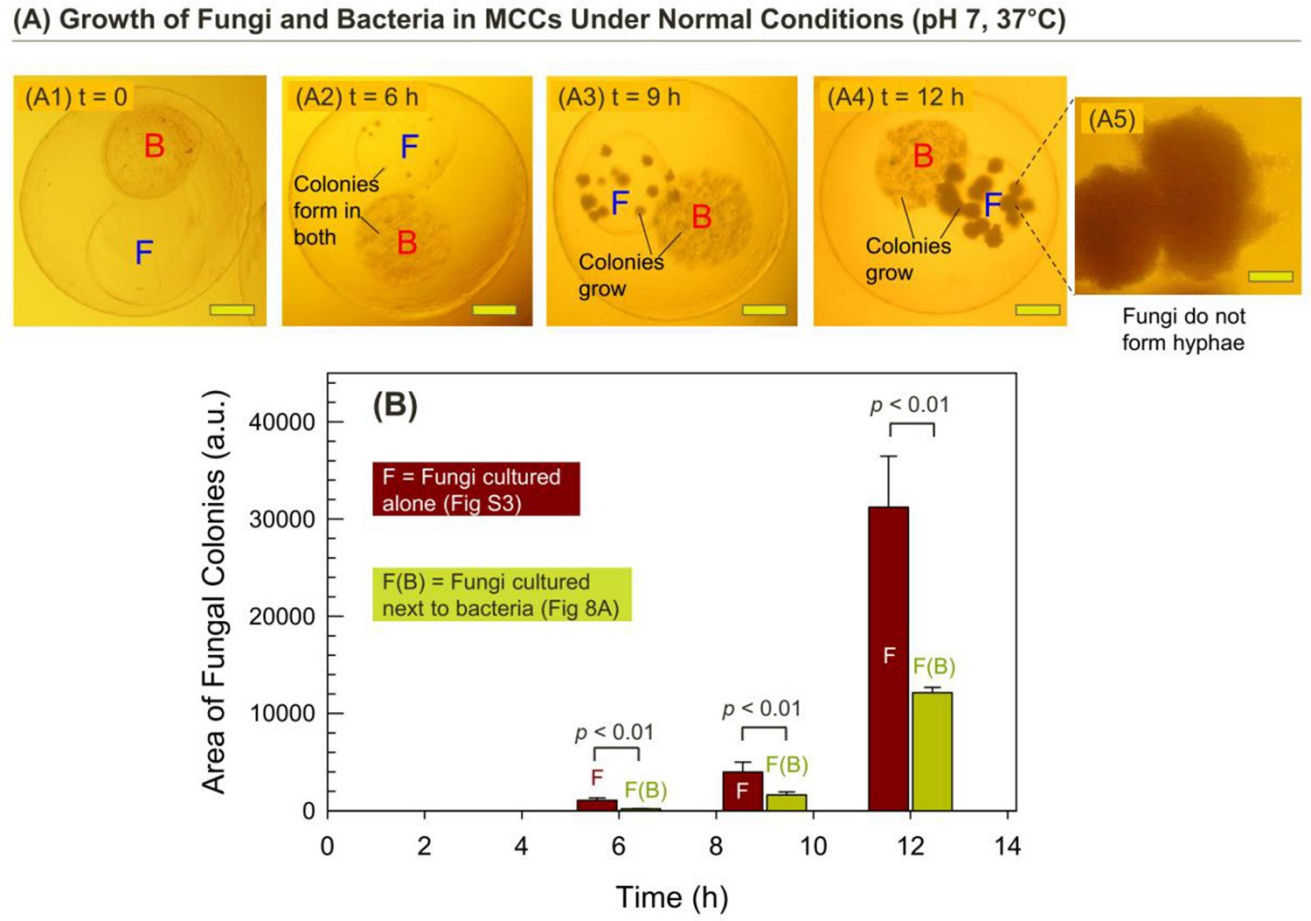
Crosstalk between *C*. *albicans* and *P*. *aeruginosa* encapsulated in distinct compartments of an MCC. (A) Optical micrographs at various time points of an MCC with Compartment B containing bacteria (*P*. *aeruginosa*) and Compartment F with fungi (*C*. *albicans*)cultured under normal conditions (37°C and pH 7). Over 12 h, both cells form colonies in their individual compartments. The fungi do not exhibit hyphae, which is attributed to the diffusion of AI-1 from the bacterial compartment. (B) A graph showing the areas covered by fungal colonies at various time points. Here F refers to the fungi cultured alone (images shown in S1 Fig in S1 File). F(B) refers to the images in (A), where the fungi are next to the bacteria. In the F(B) case, the fungal colonies pervade less area, indicating that the bacteria inhibit the fungi. The error bars correspond to standard deviations from *n* = 10 observations. (Scale bars in the images: 100 μm in A1 to A4 and 20 μm in A5.).

Next, we studied the interaction between *P*. *aeruginosa* and *C*. *albicans* using MCCs. We created MCCs with the above bacteria in Compartment B and the fungi in Compartment F. These were cultured at 37°C under normal growth conditions, and the images in Fig 8 are of a typical MCC at various time points. At *t* = 0 (Image A1), there are very few cells in both compartments, and we can differentiate Compartment B by its darker hue due to the CB nanoparticles in it. As the bacteria grow in Compartment B, they start to produce AI-1. The AI-1 molecules (size < 1 nm) are small enough to be able to diffuse out of Compartment B into the adjacent Compartment F. After 6 h, colonies of cells are seen in both compartments (Image A2). With more time, the colonies grow larger, as seen in Image A3 (9 h) and Image A4 (12 h). Importantly, the fungal colonies retain a spheroidal shape without visible hyphae around their surfaces, as shown also by the close-up in Image A5. This is evidently because the AI-1 produced in Compartment B inhibits the yeast-to-hyphae transition in Compartment F. Thus, the MCC experiment directly reveals the cross-talk between the two kingdoms of cells.

We also measured the areas of *C*. *albicans* colonies at various time points in Fig 8A using the same image-analysis procedure used previously in Fig 3. The results (Fig 8B, green bars) show a small increase in colony area from 0 to 9 h and then a sharp increase in this area from 9 to 12 h. As in Fig 3, once the colonies form, the number of colonies does not increase much, but each colony grows larger. To confirm that the fungi are indeed affected by the bacteria, we ran the same experiment with just the fungi in a compartment of the MCC but no adjacent bacteria. From the results (see S1 Fig in S1 File), we again calculated the areas of fungal colonies, and these are also plotted in Fig 8B (brown bars). The brown bars are much higher than the green ones, indicating that the *C*. *albicans* colonies grow to a lesser extent when the bacteria are present. In other words, the *C*. *albicans* is inhibited by the *P*. *aeruginosa*. S1 Fig also confirms that, in the absence of the bacteria the fungi are in the hyphal form.

We then wanted to test our hypothesis that the inhibitory action of *P*. *aeruginosa* was due to their secreting AI-1. For this, we measured how much AI-1 was present in the culture media after 12 h of culture in a sample with 50 MCCs (each with B and F compartments, as shown in Fig 8). We used a reporter strain of *E*. *coli* that produce bioluminescence proportional to the AI-1 present (see Experimental Section for details) [37]. From this assay, we measured the AI-1 concentration in the culture media to be 19 μM. Based on Fig 7, this concentration of AI-1 is sufficient to inhibit hyphal growth of the encapsulated fungi. Also, because of the proximity between the bacteria and fungi in the MCCs, the local concentration of AI-1 may have been even larger [38].

As a final observation, we also note that the cross-talk between fungi and bacteria works in both directions. Not only do the bacteria inhibit the fungi, but the fungi also affect the response of the bacteria. Specifically, production of the pigment pyocyanin by *P*. *aeruginosa* is inhibited by the presence of *C*. *albicans* [39], as shown by the results in S2 Fig in S1 File. The reduction in pyocyanin is due to the effect of farnesol, a QS molecule secreted by *C*. *albicans*. Farnesol represses genes in *P*. *aeruginosa* that trigger the production of pyocyanin [39]. While this was not a focus of our study, it is still interesting to observe, and could be studied in detail using MCCs in the future.

## 3. Conclusions

In this study, we have demonstrated that MCCs can be used as a convenient platform for studying microbes from different kingdoms in a systematic and simultaneous manner. The model microbes we chose to study were *P*. *aeruginosa* (bacteria) and *C*. *albicans* (fungi), which are known to co-exist in biofilms. We created MCCs with the bacteria and fungi in distinct inner compartments. The cells mostly grew within their compartments over the 12 h culture period. We examined conditions where one of the microbes grew preferentially compared to the other. In the presence of kanamycin or at acidic pH, only the fungi grew. Conversely, in the presence of a cationic surfactant (CTAB), only the bacteria grew. These model studies demonstrate how our MCC platform could be used in future studies to evaluate new antimicrobial compounds. Using optical microscopy, we can quantify whether both microbial kingdoms are equally affected by the compound or if the effects are mostly felt by one kind of microbe.

The MCC platform also allows us to visualize in real-time any changes in the morphology of the microbes caused by added compounds or by varying the external conditions. Under normal growth conditions, both the fungi and bacteria grew in the MCCs, but the morphological transition of *C*. *albicans* from yeast to hyphae was inhibited by AI-1, which is a QS molecule secreted by *P*. *aeruginosa* in the adjacent compartment. These model studies demonstrate how MCCs can be used to study cross-kingdom communication between encapsulated cells in discrete compartments.

The MCC platform has many advantages over current culture methods. In liquid co-culture, cells are in physical contact during their growth, which can affect their growth rates. On an agar plate, cells grow in 2-D rather than in 3-D. In the case of MCCs, each kind of cells grow within their own compartment, and thus do not come in physical contact with their opposing counterpart. Moreover, the hydrogel scaffold in both compartments offers a 3-D environment for cell growth. For these reasons, we anticipate the MCC to grow in popularity as a simple, yet versatile platform for interrogating microbes of different kinds.

## 4. Experimental section

### 4.1. Materials

The following chemicals were obtained from Sigma-Aldrich: alginate (medium-viscosity alginic acid, sodium salt from brown algae), oligochitosan (chitosan oligosaccharide lactate, molecular weight of 5,000 Da), calcium chloride dihydrate (CaCl_2_), sodium acetate, phosphate buffered saline (PBS), glutaraldehyde (a 50% solution in water), and N-(3-oxododecanoyl)-L-homoserine lactone (AI-1). Yeast extract peptone dextrose (YPD) and Luria broth (LB) media were from Life Technologies. Kanamycin and the cationic surfactant cetyl trimethylammonium bromide (CTAB) were obtained from ThermoFisher. Carbon black (CB) particles (N110) were from Sid Richardson Carbon Company.

### 4.2. Organisms and culture conditions

*P*. *aeruginosa* (PAO1), a clinical isolate, was used throughout this study [40]. *C*. *albicans* (SC5314) was purchased from American Type Culture Collection (ATCC) [41]. *P*. *aeruginosa* cells were inoculated from a frozen stock into 5 mL of YPD liquid medium. *C*. *albicans* cells were inoculated from a YPD agar plate (1% yeast extract, 2% peptone, 2% glucose, and 2% agar) into 7 mL of YPD liquid medium. Both microbes were grown overnight at 30°C while shaking at 250 rpm.

### 4.3. Growth curves

Growth curves of the fungi and bacteria in the presence of CTAB (500 μM) were recorded as the cells grew in flasks placed on an incubator-shaker at 37°C and 250 rpm. The optical density at 600 nm (OD_600_) was measured every 30 min for 6 h using a Nanodrop ND-1000 spectrophotometer.

### 4.4. Preparation of cell-bearing microcapsules

Overnight cultures of *P*. *aeruginosa* and *C*. *albicans* were subcultured separately into 5 mL of YPD at 1:100 dilution and grown to an OD_600_ of 0.4 at 30°C while shaking at 250 rpm. Next, 5 mL of each culture was spun down at 4°C, 2000 rpm for 20 min. Cell pellets were re-suspended in 800 μL of PBS and 200 μL of YPD to reach a final OD_600_ of 0.2. Cell-bearing microcapsules were prepared by the microfluidic technique described under Fig 2 and discussed in further detail in our earlier paper [11]. For this, a feed solution was prepared by mixing 200 μL of the *P*. *aeruginosa* culture with 1.8 mL of 2% alginate dissolved in PBS. Dilute concentrations of CB particles (0.01%) were added to the feed for staining the final structures. The feed was sent at 10 μL/min through a 150 μm diameter capillary. Droplets were formed at the capillary tip by pulsing nitrogen gas at 1 Hz, with the gas pressure at 7 psi. The droplets were converted to microcapsules when introduced into the reservoir solution containing 0.1 M CaCl_2_ and 1 wt% oligochitosan. After collection for 1 h, the microcapsules containing bacteria were washed three times with cold PBS and resuspended in 10 mL of PBS on ice. The same procedure was repeated with the *C*. *albicans* culture to prepare microcapsules containing the fungal cells (no CB was added to the feed in this case).

### 4.5. Preparation of MCCs with bacteria and fungi

MCCs were prepared by the same microfluidic technique described under Fig 2 and detailed in our earlier paper [11]. For this, microcapsules with *P*. *aeruginosa* and *C*. *albicans* cells (each in PBS) were mixed in a 1:1 ratio. 1 mL of this mixture was combined with 4 mL of 2% alginate dissolved in PBS. This mixture was fed through a 400 μm glass capillary at a flow rate of 40 μL/min. Droplets were formed at the capillary tip by pulsing nictrogen gas at 1 Hz, with the gas pressure at 10 psi. The droplets were collected in the reservoir containing 0.1 M CaCl_2_ and 1 wt% oligochitosan, whereupon the droplets were converted to MCCs. After collection for 30 min on ice, the MCCs were washed three times with PBS and then resuspended in PBS.

### 4.6. Culture of cells in the capsules

MCCs with both bacteria and fungi inside the inner compartments were visually identified and taken out from the batch to be placed in a well of a 12-well plate using a transfer pipette. The MCCs were incubated at 37°C in YPD medium while being shaken at 250 rpm. Culture was typically done for 12 h.

### 4.7. Measurement of AI-1 produced by the encapsulated bacteria

A bioluminescent reporter assay was used to measure the AI-1 produced by *P*. *aeruginosa* in the microcapsules [37]. The microcapsules were incubated for 18 h and the surrounding media was collected, filtered through a 0.2 μM filter, and stored at −20°C until needed. Luminescent *E*. *coli* reporter cells with the pAL10545 plasmid were grown in LB media overnight. The next day, the cells were diluted 2500-fold in LB media with 50 μg/mL tetracycline and 50 μg/mL kanamycin. The stored media samples were diluted in LB to be within the linear range of the assay. Samples for a standard curve of known AI-1 concentrations ranging from 0–60 nM AI-1 in LB were also prepared. 10 μL of the experimental or standard-curve samples were added to 90 μL of the reporter cells. Cultures were grown at 30°C and 250 rpm shaking, and luminescence values were recorded after 3 h using a Promega GloMax®-Multi Jr plate reader. Each measurement was performed in triplicate.

### 4.8. Optical microscopy

Brightfield images of the microcapsules were obtained using an inverted optical microscope (Zeiss Axiovert 135 TV) using a 2.5× objective.

### 4.9. Measurement of colony areas

The area of fungal colonies inside MCCs were analyzed from the bright-field images by ImageJ with a Colony Counter plug-in. The Colony Counter separated each colony from the background image and reported the area in arbitrary units (a.u.). In case of overlapping colonies, the combined area was calculated and divided by the total number of overlapping colonies. Areas of fungal colonies inside at least 10 MCCs were averaged and are reported in Figs 3 and 8 at various time points.

### 4.10. Scanning Electron Microscopy (SEM)

To prepare samples for SEM, alginate microcapsules bearing *C*. *albicans* cells were dried using the method described by Suvarna *et al* [42]. Briefly, microcapsules were kept in 2 wt% glutaraldehyde in PBS at 4°C for 3 h. The capsules were then washed in a series of ethanol washes (50, 70, 90, 100%) for 15 min at each dilution and dried overnight at room temperature. The dried capsules were pipetted onto a double-sided carbon tape that in turn was attached on an SEM stub. The samples were coated with gold and examined on a Tescan XEIA FEG SEM with an accelerating voltage of 5 kV.

### 4.11. Statistical analysis

Data are represented as mean ± standard deviation. Statistical difference between experimental groups was evaluated using a two-tailed student’s t-test. Results were considered to be statistically significant when a *p*-value was less than 0.05.

## Supporting information

S1 File (PDF)
