# Supplementary material for: Capsules with bacteria and fungi in distinct compartments: A platform for studying microbes from different kingdoms and their cross-communication
Source: PLoS One. 2022 Nov 11;17(11):e0277132. doi: 10.1371/journal.pone.0277132 (PMC9651590; doi:10.1371/journal.pone.0277132)
Supplement: S1 File — (PDF) [file pone.0277132.s001.pdf]

Supporting Information for:

Capsules with Bacteria and Fungi in Distinct Compartments: A Platform for Studying Microbes from Different Kingdoms and their Cross-Communication

So Hyun Ahn,[1] Amy J. Karlsson,[1,2] William E. Bentley[1,2]* and Srinivasa R. Raghavan[1,2]*

[1]Department of Chemical & Biomolecular Engineering, University of Maryland, College Park, Maryland 20742, USA

[2]Fischell Department of Bioengineering, University of Maryland, College Park, Maryland 20742, USA

*Corresponding authors. Emails: sraghava@umd.edu, bentley@umd.edu

Contents

Figure S1: Growth of fungi in an MCC under normal conditions.

Figure S2: Fungi inhibit bacterial production of pyocyanin.

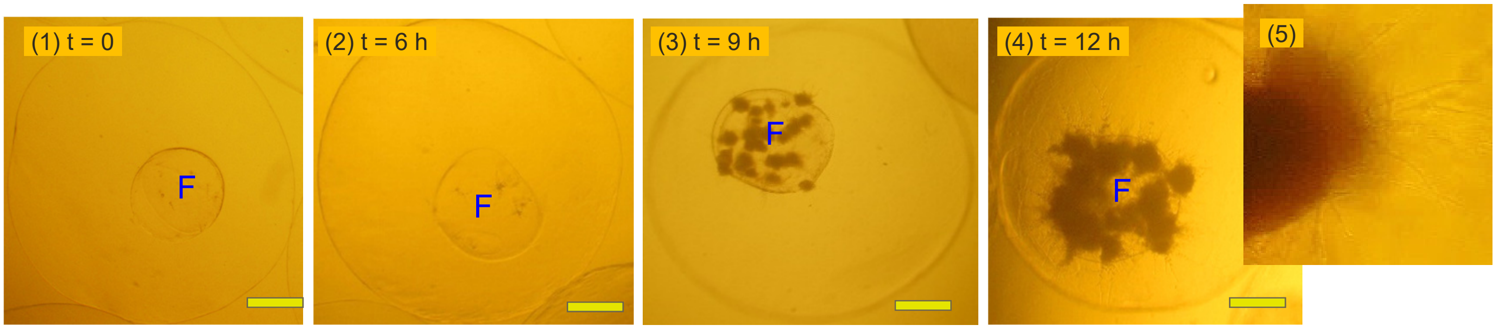

Figure S1. Growth of fungi in an MCC under normal conditions. The MCC has a single compartment containing fungi (*C. albicans*) and is cultured under normal conditions (37°C and pH 7). This experiment complements the one shown in Figure 8A, where the MCC has compartments of both fungi and bacteria. Over 12 h, the fungi form colonies and exhibit hyphae at the colony edges, as shown by the close-up image (5). The areas covered by fungal colonies at various time points are computed from the images and are plotted in Figure 8B. Scale bars in the images: 100 μm in (1) to (4) and 20 μm in (5).

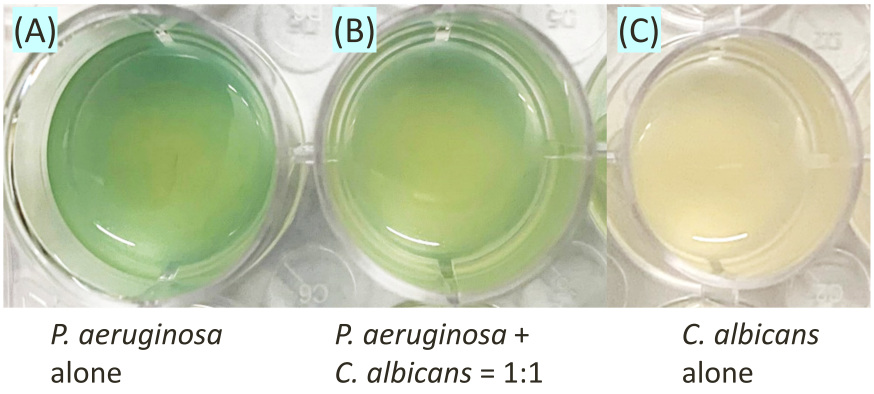

Figure S2. Fungi inhibit bacterial production of pyocyanin. (A) Capsules containing bacteria (*P. aeruginosa),* cultured for 12 h, give the media a blue/green color due to production of the pigment pyocyanin. (B) The same number of capsules with the bacteria are combined with an equal number of capsules containing the fungi (*C. albicans*). Less color is visible after 12 h of culture, indicating lower pyocyanin in the media. This suggests that the fungi inhibit the bacterial production of pyocyanin (C) As a control, capsules with the fungi alone do not change the color of the media after 12 h of culture, as the fungi do not produce pyocyanin.
